# The effect of widowhood on depression of caregivers

**DOI:** 10.1186/s12913-023-09746-4

**Published:** 2023-07-03

**Authors:** Jiahui Pang, Dachuan Liang, Yuanyang Wu

**Affiliations:** 1 School of Public Administration, Zhongnan University of Economics and Law, Wuhan, 430073 China; 2 The Linfen People’s hospital, The Affiliated Linfen Hospital of Shanxi Medical University, Linfen Shanxi, 041000 China; 3 School of Medicine and Health Management, Tongji Medical College, Huazhong University of Science and Technology, Wuhan, 430030 Hubei China

**Keywords:** Widowhood, Caregivers, Depression, Channel analysis, Propensity score matching

## Abstract

**Background:**

It is known that caregivers are more likely to be depressed compared to those without caregiving burden. The disappearance of caregiving burden after widowhood may alleviate depression, but at the same time the diminishment of marital resources caused by widowhood may exacerbate depression. So, what effect does widowhood have on depression among caregivers?, which was valuable for promoting the mental health of caregivers in the context of China’s aging.

**Methods:**

China Health and Retirement Longitudinal Study (CHARLS), a longitudinal data, was selected and the effect of widowhood on depression among middle-aged and elderly caregivers was explored by using Ordinary Least Squares and Propensity Score Matching methods based on 2018 CHARLS data. The channels and subgroup difference were also analyzed.

**Results:**

Widowhood significantly increased CES-D scores of caregivers, and there were higher CES-D scores among women, the middle-aged, rural residents, and those with higher levels of education. Widowhood increased depression of caregivers through reducing personal economic resources, and increasing possibilities to live with children and participate in social activities.

**Conclusions:**

Caregivers who experienced widowhood tend to be depressed and concerted efforts are needed. On the one hand, more social security measures and economic subsidy policy should focus on the middle-aged adults and elderly who experienced widowhood. On the other one hand, it is helpful to relieve depression by providing more social support from society and families to the middle-aged adults and elderly who experienced widowhood.

**Supplementary Information:**

The online version contains supplementary material available at 10.1186/s12913-023-09746-4.

## Introduction

With the rapid advancement of aging, there were approximately 6.19 million disabled older adults in 2020, and it was predicted that the scale would reach 8.95 million in 2030, 10.43 million in 2040, and 11.52 million in 2050 in China. If the disease factors are considered, the scale would almost double in the next four decades [1]. Once an older adult is disabled, at least one person in the family needs to provide care for the older adult who is unable to do activities of daily living [2]. The caregivers in family is exposed to caregiving stress, financial burdens, physical exhaustion, and mental stress when providing long-term care. For example, people who provide care for the disabled spouses have more depressive symptoms than those without caregiving burden [3].

Widowhood is another common stressful event faced by Chinese older adults [4]. Some studies demonstrated the positive effect of marriage in terms of health, which was affected by factors, such as family role patterns and emotional quality of the marriage [5, 6]. Marital resource theory indicated that spouses provide financial income, spiritual companionship and social support to the family, while the positive effect disappears after widowhood, showing a “widowhood effect” [7]. It is worth considering whether the widowhood effect still exists for caregivers. From an egoistic perspective, when the disabled dies, the spouse no longer pays for the disabled person’s medical expenses, and faces the time constraints, physical exhaustion, and mental stress [8]. However, based on a family collectivist perspective, the positive utility from marriage disappears after widowhood, which means that family resources, social support, and family resilience to risk are reduced after widowhood [9].

In China, family relationships deeply affect individual health. The disappearance of caregiving burden after widowhood may alleviate depression, but at the same time the disappearance of marital resources may exacerbate depression. So, what effect does widowhood have on depression among caregivers?, which was valuable for promoting the mental health of caregivers in the context of China’s aging. Therefore, we aimed to explore the effect of widowhood on depression of caregivers and further analyze group heterogeneity and its channel.

## Literature review and theoretical framework

### Literature review

Large number of studies have demonstrated that widowhood increased depression of older adults [10–14]. In China, it found that scores on the geriatric depression scale (GDS) were higher in the widowhood group, and the detection rate of depression in the widowed group was 29.8%, about 2.2 times higher than that in the elderly with spouse [15]. Widowhood significantly increased depression of older adults [11]. By using the PSM-DID method, it further found that widowhood significantly increased depression among the elderly who were females, lived in rural areas, and with low education attainment [16]. Sun and Zhang et al. found that lower mental health scores for widowed older adults than those with a spouse by using subjective well-being as a proxy for mental health [17]. In conclusion, widowhood, as a common event in later life, leads to a high risk of depression, poor physical health and high risk of death [18].

Economic status and social network were possible channels in the association between widowhood and caregivers’ depression. Loss of social support, and poor economic resources after widowhood had a negative effect on physical and mental health in later life [7, 9, 16]. It has been found that both individual and family social capital after widowhood were lower than those with a spouse, and the mental health became better when increasing social capital [19]. There is a correlation between personal social capital and loneliness among widowed elderly, and the rural widowed elderly had higher loneliness than urban because of lower social capital [20]. At the same time, higher pension benefits could weaken the adverse effects of widowhood [21]. In addition, some studies found that family networks mediated the relationship between widowhood and depression from the perspective of family network and social network, while social networks masked the relationship [11]. And the mental health could be improved by more daughters and neighborhood’s interactions, and frequent relative interactions [17].

Similarly, caregiving also increased depression of caregivers [2, 22, 23]. Providing in-home care for the disabled increased the center for epidemiological studies depression scale (CES-D) scores by 10% [2], and the probability of depression among rural caregivers was 49.4%, which was higher than the 41.7% in urban areas [24]. There was a poorer mental health for those who provided caregiving compared to those who did not [25]. Several studies focusing on older caregivers have found that older caregivers are more depressed than the older adults without caregiving [26], which was associated with changes in social relationships in response to role transitions [27].

### Theoretical framework

**Fig. 1:**
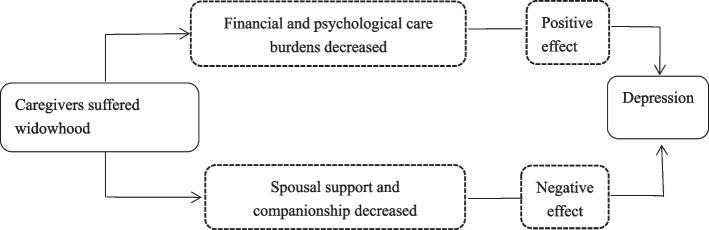
Pathways of widowhood on depression of caregivers

Stress process model explained why providing caregiving impaired caregivers’ health [2, 22, 25, 28]. Family caregiving was persistent, uncontrollable, unpredictable, and created physical and psychological stress [29]. It found that participating in basic pension insurance reduced caregivers’ CES-D scores, and pension insurance smoothed out the negative effects of caregiving on mental health [2]. And further studies showed that providing support to caregivers, living with children, participating in social activities and personal economic resources weakened the negative effect of caregiving [25]. Additionally, social support and self-efficacy significantly reduced depression of caregivers [30]. Based on above studies, in Chinese context, caregivers’ depression may be relieved after widowhood due to the disappearance of caregiving burden, but the depression may also be exacerbated by the disappearance of marital resources. Therefore, dose widowhood reduce the burden of caregivers or increase the depression of caregivers? The channel was shown in Fig. 1. The association is few explored in China and needs to be supported by further empirical evidence.

## Methods

### Samples

Four hundred sixty-six caregivers who have experienced widowhood from China Health and Retirement Longitudinal Study (CHARLS) in 2018 cross-sectional data were selected for this study, which was a large micro-survey data of people aged 45 and above hosted by Peking University in China. The reason why we selected 2018 cross-sectional data was representative and timeliness, which was a nationwide survey and latest available data. 466 caregivers were identified by following steps: CHARLS team randomly selected a family member aged over 45 years from each household as the main interviewee. So, first, 2015 CHARLS data was used to identify households with one disabled person who received spouse care. Second, the data was matched with 2018 CHARLS data. Thirdly, we identified whether the disabled person died in the matched data, indicating whether the caregiver suffered from widowhood. Finally, 466 caregivers were retained after excluding outliers and missing values.

### Ethics

The CHARLS was approved by the Ethics Committee of Peking University Health Science Center. Written informed consent was obtained from each participant.

### Variables

#### Outcome

The Center for Epidemiological Studies Depression Scale (CES-D) scores was selected to measure the mental health as the outcome. Referring to the existing literature [31, 32] and the CHARLS questionnaire, the CES-D, was selected it to measure individual depression status. 2018 CHARLS questionnaire questions contain 10 items of the depression, among which 8 items of negative emotions included “bothered by small things”, “have difficulty concentrating on things”, “Feeling depressed”, “Feeling hard to do everything”, “Feeling afraid”, “Poor sleep”, “Feeling lonely” and “Feel unable to continue living”, and two positive emotions “hopeful for the future” and “very pleasant”. Respondents’ responses to the question on negative emotions were rated 0 ~ 3 on a scale of “rarely or not at all”, “not very much”, “sometimes or half of the time”, and “most of the time”. Respondents with positive emotions were reverse coded and assigned a score of 3–0. The CES-D were summed, and the higher scores indicated more depressions. The total score less than 10 was defined as non-depression with a value of 0, and the total score more than and equal to 10 was defined as depression with a value of 1.

In addition, other outcome variables were selected for identifying the transmission channel, including personal assets, living with children, and participating in social activities, which aimed to further explore the causal relationship and the transmission mechanism between the widowhood and depression. *Personal assets* were the sum of cash, deposits, bonds, stocks and funds, which were set as a continuous variable in logarithmic form. *Living with children* was a dummy variable indicating whether the caregiver lived with their children, with 1:yes, 0:no. *Participating in social activities* was also a dummy variable indicating whether the caregiver participated the social activities, with 1:yes, 0:none.

#### Explanatory variables

Widowhood was the core explanatory variable, indicating the death of individual who received care in the family. We identified the individual’s death by “death or not” in CHARLS data, and generated a dummy variable, indicating whether the individual suffered a widowhood, with 1 of suffering widowhood and 0 of not.

#### Control variables

Age, gender, living area, education level and number of chronic diseases were associated with depression [2, 11]. Combined with existing research, age, gender, living area, education level, number of chronic diseases, region^1^ and ethnic group were selected as the control variables. Gender, living area, education level, region and ethnic group were dummy variables, and age and number of chronic diseases were continuous variables in Table 1.

**Table 1:** Basic descriptive statistics

Variables	Variable Definition	Widowed			Non-widowed			T-test
		N	Mean	Std	N	Mean	Std	MeanDiff
Depression	0 = not depressed; 1 = depressed	77	0.55	0.50	389	0.42	0.49	-0.124**
Gender	0 = female; 1 = male	77	0.32	0.47	389	0.52	0.50	0.200***
Age		77	70.10	9.50	389	65.77	8.97	-4.346***
living area	0 = urban; 1 = rural	77	0.82	0.39	389	0.78	0.42	-0.039
Education level	0 = illiterate; 1 = elementary school and below; 2 = junior high school and above	77	0.88	0.74	389	1.06	0.68	0.179**
Number of chronic diseases^a^	Number of diseases, ranged from1 ~ 14	77	2.37	1.84	389	2.72	2.16	0.343
Region	0 = north; 1 = south	77	0.34	0.48	389	0.36	0.48	0.020
Ethnic group	0 = Han ethnic group; 1 = other ethnic groups	77	0.12	0.32	389	0.07	0.26	-0.042
Personal assets	Log of personal assets	77	5.25	3.45	389	6.29	3.47	1.032**
Living with children	0 = no; 1 = yes	77	0.45	0.50	389	0.30	0.46	-0.154***
Participating in social activities	0 = none; 1 = yes	77	0.60	0.50	389	0.49	0.50	-0.104*

^a^ “Number of chronic diseases” indicated the number of diseases a caregivers had, ranged from 0 ~ 14. The scope of chronic diseases in this paper was based on the classification criteria of the adopted questionnaire of the CHARLS database, which classified chronic diseases into 14 categories, as follows: Hypertension; Dyslipidemia (elevation of low density lipoprotein, triglycerides (TGs), and total cholesterol, or a low high density lipoprotein level); Diabetes or high blood sugar; Cancer or malignant tumor (excluding minor skin cancers);Chronic lung diseases, such as chronic bronchitis, emphysema (excluding tumors,or cancer); Liver disease (except fatty liver, tumors, and cancer); Heart attack,coronary heart disease, angina, congestive heart failure, or other heart problems; Stroke; Kidney disease (except for tumor or cancer); Stomach or other digestive diseases (except for tumor or cancer); Emotional, nervous, or psychiatric problems; Memory-related disease (such as dementia, brain atrophy, and Parkinson’s disease); Arthritis or rheumatism; Asthma. In this paper, each type of chronic disease was assigned a value of 1 and no disease was assigned a value of 0. The number of chronic diseases was defined as the sum of the types of chronic diseases, and the range of assignment was 0–14

*p < 0.010, **p < 0.050, ***p < 0.001

### Statistical strategy

#### Basic regression model

Ordinary Least Squares regression model was performed to analyze the effect of widowhood on depression of caregivers. On the one hand, widowhood was an exogenous shock event, and there was little endogeneity in the association. On the other one hand, the OLS result was a benchmark, providing preliminary identification of the association. The model was as follows:

1 $${cesd}_{i}= \alpha + \beta\,{Marital\_status}_{i}+ \gamma {x}_{i}+ {\varepsilon }_{i}$$

where $cesd_{i}$ represented the CES-D scores of individual $i$, $$Marital\_status_{\begin{subarray}{l} i \\ \end{subarray} }$$ represented the marital status of individual $i$, $x{}_{i}$ represented the control variables of individual $i$, $\varepsilon_{i}$ represented the random error term, $\alpha$ represented the intercept term, and $\beta$ and $\gamma$ represented the regression coefficients of control variables.

#### Propensity score matching

Propensity score matching was to process the observable information of multiple individual characteristics into a propensity score by the Probit or Logit method, and matched the treatment and control groups in the sample based on the propensity score, and calculated the average treatment effect (ATT) of the participants. This was set in the following equation:

2 $$ATT = E[ces - d_{1i} |D_{i} = 1,p(X_{i} )] - E[ces - d_{0i} |D_{i} = 0,p(X_{i} )]$$

where $ces - d_{i}$ was CES-D scores; $D_{i}$ was the treatment variable, mainly referring to $widow_{i}$, $D_{i} = 1$ when it indicated widowhood and $D_{i} = 0$ when it indicated non-widowhood; $p(X_{i} )$ was the propensity score indicating the probability of widowhood, which was estimated by a logit model.

#### Channel analysis

There was many studies carrying channel analysis when exploring the causal relationship [10, 33, 34]. The detailed channel analysis was performed as follows: first, the causality between channel variables and explanatory variables was analyzed based on theoretical perspective rather than formal causal inference instruments. Second, the regression analysis of the explanatory variable on the channel variable was needed [35].

The outcome in Eq. (1) were replaced with three channel variables: personal assets, living with children, and participating in social activities, referring to existing studies approach [10, 33]. The model was set as follows:

3 $$Mechanism_i=\alpha_1+\beta_1{Marital\_status}_i+\gamma_1x_i+\varepsilon_i$$

where $Mechanism_{i}$ were the channel variables, including personal assets, living with children and participating in social activities. If $\beta_{1}$ significant, we considered it as a channel variable. The other variables were defined in the same way as in Eq. (1).

## Results

### Descriptive results

83.3% of the individuals were not widowed and 16.7% were widowed. Firstly, in terms of CES-D scores, the mean scores of the widowed group were higher than that non-widowed group. Secondly, compared to the non-widowed group, the widowed group was older, female, and had lower level of education. There were almost no differences between the widowed and non-widowed groups in terms of living area, number of chronic diseases, region and ethnic group, and a high proportion of rural households, northern and Han ethnic group, and about 2 or 3 chronic disease co-morbidities. The mean of personal assets was lower for the widowed group than for the non-widowed group, and the probability of living with children and participating in social activities were higher than for the non-widowed group.

**Fig. 2:**
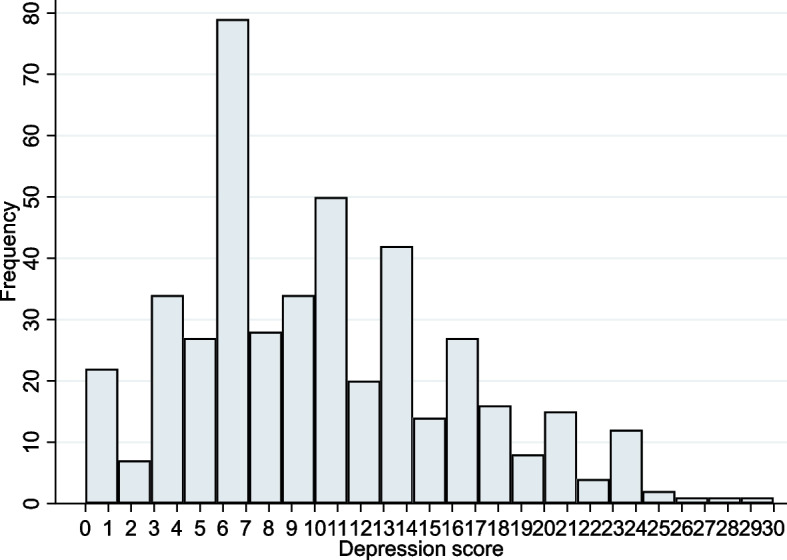
Depression score chart for caregivers in 2015

**Fig. 3:**
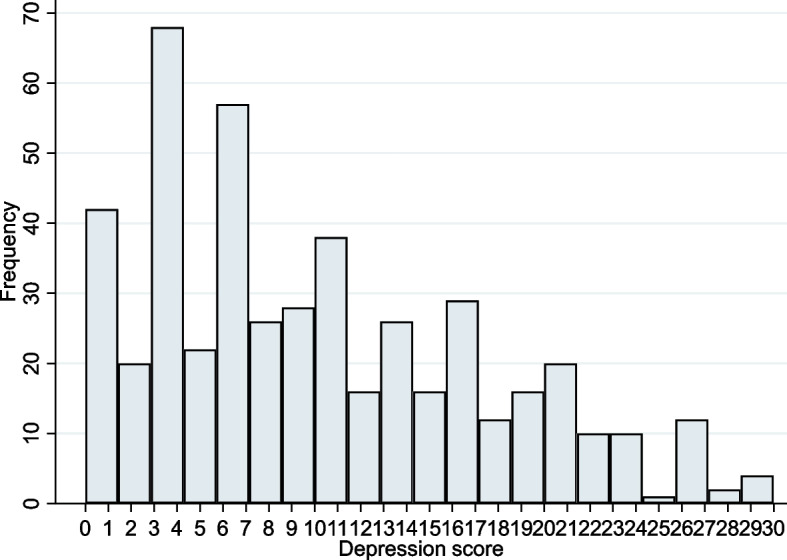
Depression score chart for caregivers in 2018

Comparing the depression scores of caregivers in 2015 and 2018, it was clear that there were more high depression scores in 2018 than in 2015 from the Figs. 2 and 3.

### Baseline regression results

Table 2 showed the effect of widowhood on depression of caregivers in model 1 and 2. It found that the CES-D scores of caregivers increased significantly after widowhood, which was consistent with existing research. For example, some studies found that the negative effect of widowhood on the mental health of caregivers did exist after excluding differences in the sample [8].

**Table 2:** Effect of widowhood on CES-D scores of caregivers

	Model(1)	Model(2)
*Variables*	Depression	Depression
*Widowhood (1* = *widowed)*	0.124**	0.138**
	(0.062)	(0.059)
*Gender (1* = *male)*		-0.091**
		(0.046)
*Age*		-0.005*
		(0.003)
*Living area (1* = *rural)*		0.024
		(0.058)
*Region (1* = *south)*		0.086*
		(0.046)
*Ethnic group (1* = Other ethnic groups*)*		-0.165**
		(0.079)
*Education level*		-0.121***
		(0.036)
*Number of chronic diseases*		0.075***
		(0.009)
*_cons*	0.422***	0.662***
	(0.025)	(0.204)
*N*	466	466

①*p < 0.010, ** *p* < 0.050, *** *p* < 0.001

②standard errors were in parentheses

In addition, control variables were associated with depression. Compared to the males, female caregivers had a higher CES-D scores after widowhood, which was consistent with the existing research findings [16, 25]. Caregivers in the south were more likely to be depressed after widowhood. Higher education attainment was beneficial to decrease the CES-D scores among older adults, which was consistent with the existing research finding [11]. More chronic diseases also were associated with higher CES-D scores, which was consistent with existing studies [36].

### Robustness checks

#### PSM results

When estimating the effect of widowhood on depression, there was a selection bias, which means that some individuals have depression before the widowhood occurs, resulting a biased estimation. The propensity score matching method could effectively overcome the estimation bias from individual observable factors and thus achieved valid estimates. Matching similar individuals enables estimation among similar individuals, thus obtaining the net effect of widowhood on depression. Therefore, PSM was further used for robustness tests, and the results were shown in Table 3. The results showed that widowhood significantly increased CES-D scores of caregivers, showing a significant negative effect on mental health, which was consistent with the basic regression results.

**Table 3:** Average treatment effect of widowhood on depression of caregivers

	Sample	Treatment group	Control group	ATT	SD	t
1vs10	Before	0.545	0.422	0.124	0.062	2.000
	After	0.553	0.386	0.166^**^	0.069	2.420
Radius Matching	Before	0.545	0.422	0.124	0.062	2.000
	After	0.562	0.380	0.182^**^	0.069	2.620

^*^p < 0.010, **p < 0.050, ***p < 0.001

#### Validity identification test

**Fig. 4:**
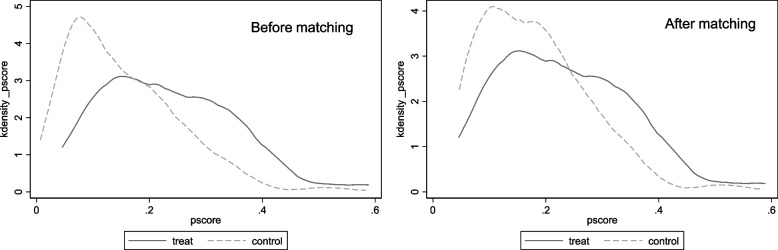
Common support test

Common support assumptions and data balance tests were required to ensure the valid estimation. The results were shown in Fig. 4 and Table 4. After matching, the kernel density functions in Fig. 4 had more overlapping areas and decreased in kurtosis, which achieved better co-support. The standard deviation of each variable in Table 4 decreased and *P* > 0.1, which satisfied the requirement of data balance.

**Table 4:** Data balance test before and after matching

Variables	Sample	Average value		SD%	t-test	
		Treatment group	Control group		t	P >|t|
Gender	Before Matching	0.325	0.525	99.8	-3.23	0.001
	After matching	0.329	0.330		-0.01	0.996
Age	Before Matching	70.117	65.771	94.9	3.85	0.000
	After matching	70.026	69.803		0.16	0.874
living area	Before Matching	0.818	0.779	92.7	0.77	0.444
	After matching	0.816	0.819		-0.05	0.964
Region	Before Matching	0.338	0.357	-158.8	-0.33	0.742
	After matching	0.329	0.380		-0.65	0.515
Ethnic group	Before Matching	0.117	0.075	54.2	1.24	0.216
	After matching	0.105	0.086		0.40	0.687
Education level	Before Matching	0.883	1.062	76.9	-2.08	0.038
	After matching	0.895	0.936		-0.36	0.719
Number of chronic diseases	Before Matching	2.377	2.720	70.7	-1.30	0.194
	After matching	2.408	2.374		0.33	0.745

### Heterogeneity analysis

The effect of widowhood on depression of caregivers might be influenced by individual characteristics and social and economic factors, thus showing differences among different subgroup. Age was selected for heterogeneity analysis due to different physical depreciation and opportunity cost of time for caregivers in different age group. First, *China’s Law on the Protection of the Rights and Interests of the Elderly* stipulates that the starting age standard for the elderly is 60 years old, and the elderly are usually regarded as a group with higher depreciation and lower health levels, with greater physical depletion caused by providing care. Second, the legal retirement age for male workers in China is 60 years old, and the legal retirement age for female workers is before 60 years old. The difference between retirement and employment is reflected in the lower economic income level and lower opportunity cost of time. There was a question that whether men or women suffered more from depression after becoming widowed, and other studies have shown no gender differences in the mental health consequences of widowhood [5, 6].

The gender was selected for heterogeneity analysis due to the different family roles in gender in China, such as earning money for men and family care for women. The living area was also selected for heterogeneity analysis because the long-term urban–rural structure in China leads to large differences in the economic development, medical services, and social support, which has a different effect on mental health due to different stress exposure environment. And education attainment was associated with economic status, which played an important role in mental health. So, the education was selected also. Table 5 showed that compared to the non-widowed group, widowhood significantly increased depression among the female, the middle-aged adults, rural, and higher-level education groups. From models 3 to 4, the CES-D scores of the middle-aged adults increased significantly after widowhood. The CES-D scores of women were higher than men in models 5 ~ 6 after widowhood. The results from models 7 ~ 8 and model 9 ~ 10 suggested that the CES-D scores among the rural and the higher education level group were higher than those living in urban or with low education level.

**Table 5:** The effect of widowhood on depression of caregivers among subgroup with different age, gender, living area, and education attainment

	Model (3)	Model (4)	Model (5)	Mode (6)	Model (7)	Model (8)	Model (9)	Model (10)
*Variables*	≤ 60 years	> 60 years	female	male	urban	rural	illiterate	non-illiterate
*Widowhood*	0.260*	0.098	0.142*	0.134	0.213	0.115*	0.095	0.149**
	(0.142)	(0.063)	(0.023)	(0.102)	(0.139)	(0.065)	(0.109)	(0.071)
*Control variable*	YES	YES	YES	YES	YES	YES	YES	YES
*_cons*	0.037	0.408***	0.630**	0.515*	0.341	0.744***	0.221	0.481**
	(0.181)	(0.090)	(0.293)	(0.300)	(0.411)	(0.210)	(0.507)	(0.194)
*N*	115	351	237	229	100	366	104	362

Gender, age, living area, region, ethnic group, education level, and number of chronic diseases were included as control variables

*p < 0.010, **p < 0.050, ***p < 0.001

### Channel analysis

The channels between widowhood and depression were explored through personal assets, living with children, and social activities. Table 6 showed that widowhood decreased caregivers’ personal assets, and widowhood reduced the economic resources available of individuals. However, widowhood increased the probability of living with children and the probability of participating in social activities.

**Table 6:** Channel analysis of the effect of widowhood on depression of caregivers

	Model(11)	Model(12)	Model(13)
Variables	Personal assets	Living with children	Participating in social activities
Widowhood	-0.866**	0.151**	0.156**
	(0.430)	(0.064)	(0.065)
Control variables	Yes	Yes	Yes
_cons	7.135***	0.657***	0.896***
	(1.551)	(0.194)	(0.210)
N	466	466	466

Gender, age, living area, region, ethnic group, education level, and number of chronic diseases were included as control variables

*p < 0.010, **p < 0.050, ***p < 0.001

## Discussion

### Widowhood significantly increased depression of caregivers

Widowhood significantly increased the depression of caregivers based on the above estimation. Widowhood might lead to lower economic income from marital resource theory. The survivor pension system was implemented in developed countries to buffer negative effect of the late-life widowhood, while China did not has the system. Widowhood led to poverty of older adults [21], and income could be considered as a resource advantage, which had a significant positive effect on the mental health of older adults [37]. Second, widowhood leads to a stress exposure environment and a high incidence of social isolation from the stress process theory. Social isolation was manifested by narrowing social networks and increased loneliness [38], social networks, including family and friend networks, became smaller after widowhood [11, 39].

### There were group differences in caregivers’ depression after widowhood

There were significant group differences of caregivers. Women were more likely to be depressed after widowhood because of the different psychological health status of different genders. Some studies have shown that the incidence of depression in older women in China is higher than in men, with women having a higher propensity to depression [15]. Women suffered more economic loss after widowhood based on marital resources theory [40]. The rural group was more likely to be depressed after widowhood, which was supported by existing studies. Some studies have shown that the mental health of older people in urban areas in China was usually better than that of older people in rural areas [41]. Community environment influenced the individual stress, and the difference of living in urban and rural community environments directly or indirectly affected the psychological health of older adults based on stress process theory. In China, social support, such as social security, health care, and mental recreation for older adults, was relatively weak in most rural areas [42]. The higher education level group was more likely to be depressed after widowhood, which might be explained by the fact that the relationship between education level and depression was not linear, showing a “U” shape distribution [43]. The group with higher education level had higher depression scores before widowhood, which may be explained by the more emotional needs of higher-education group.

### Widowhood decreased personal assets, but increased the probability of living with children and participating in social activities

Widowhood has a negative effect on family economic resources. In Chinese older adults, their income in later life was mainly from medical insurance^2^ and pension.^3^If widowhood happened, family economic resources decreased and the total income from pension decreased. Living with children not only alleviated the financial stress but also compensated for the lack of spiritual companionship, which had a positive effect on older adults’ health [44]. Additionally, the behavior of participating in social activities increased after widowhood. Shrinking family networks and the absence of spiritual companionship caused by widowhood could be made up with participating in social activities.

## Limitations

Despite the notable findings, this study, like most others, had limitations. First, widowhood has long-term and short-term effect [45], but this study could not identify changes affected by duration of widowhood. Some studies have shown that depression was most severe during the early stages of widowhood [10], and that post-bereaved depressive levels peaked during the first six months of widowhood and gradually diminished over 25 months [46]. It is not possible to determine the exact time limited by the widowhood events in the interval of 2015 and 2018. Secondly, due to database limitations, there were omitted variables, but no instrumental variables were found to address this problem. Based on the findings of existing studies, the omitted variables may include marital quality [5], degree of marital dependence [47], etc. Third, the data in this study is nationwide, which ignores the insufficient development between regions, and the impact of different economic status, medical resources, and social services.

## Conclusion

The effect of widowhood on depression of caregivers was investigated by using OLS and propensity score matching methods based on the 2018 cross-sectional data from China Health and Retirement Longitudinal Study (CHARLS). It found that widowhood significantly increased CES-D scores of caregivers, and this positive effect was particularly pronounced among women, middle-aged, rural residents, and those with higher levels of education. Widowhood affects depression through decreasing personal economic resources, increasing possibilities to live with children and participating in social activities.

The policy implications were, first of all, it is urgent to raise social security benefits and economic subsidy for caregivers who experience widowhood. The caregivers were faced with a poor economic status after widowhood due to the poor individual economic resources. Providing financial security for the low-income group after widowhood is beneficial to maintain caregivers’ subsequent life. Secondly, more social support is helpful for mental health of caregivers who experience widowhood, with particular attention to caregivers who are female, the middle-aged adults, rural, and with higher levels of education.

## Supplementary Information

**Additional file 1.**

## Data Availability

Data can be requested from the correspondent. The valid direct link for data accession: http://charls.pku.edu.cn/.

## Footnotes

1 CHARLS database does not contain data from Hainan, Taiwan, Hong Kong, Macao, Ningxia, and Tibet, and this study defines Guangdong, Yunnan, Guangxi, Guizhou, Jiangxi, Fujian, Jiangsu, Anhui, Hunan, Hubei, Sichuan, Chongqing, Shanghai, and Zhejiang regions were defined as the south and assigned a value of 1, and Heilongjiang, Jilin, Liaoning, Inner Mongolia, Beijing, Tianjin, Hebei, Henan, Shandong, Xinjiang Hebei, Henan, Shandong, Xinjiang, Gansu, Qinghai, Shaanxi, and Shanxi regions were defined as the north and assigned a value of 0.

2 By the end of 2017, the number of insured people in the national basic medical insurance exceeded 1.3 billion, and the coverage rate of the insured was above 95%. Data source: Zang WB, Chen C and Zhao SY. 2020. Social Health insurance, Heterogeneity of Disease and Medical Expenditures. Economic Research. 55(12):64–79.

3 By the end of 2021, the number of insured persons in the national basic pension insurance had reached 1.029 billion. Data source: Ministry of Human Resources and Social Security of the People's Republic of China:Statistical Bulletin on the Development of Human Resources and Social Security for the Year 2021,http://www.mohrss.gov.cn/SYrlzyhshbzb/zwgk/szrs/tjgb/202206/t20220607_452104.html.
