# Supplementary material for: The effect of widowhood on depression of caregivers
Source: BMC Health Serv Res. 2023 Jul 3;23:722. doi: 10.1186/s12913-023-09746-4 (PMC10316613; doi:10.1186/s12913-023-09746-4)
Supplement: Supplementary file 1 — Additional file 1. [file 12913_2023_9746_MOESM1_ESM.doc]

Supplementary tables

Supplementary table 1 Collinearity Test of variables

| variables | depression | widowhood | gender | age | Living area | Region | Ethnic group | Education level | Chronic diseases |
| --- | --- | --- | --- | --- | --- | --- | --- | --- | --- |
| depression | 1.000 |  |  |  |  |  |  |  |  |
| widowhood | 0.093 | 1.000 |  |  |  |  |  |  |  |
| gender | -0.175 | -0.148 | 1.000 |  |  |  |  |  |  |
| age | -0.004 | 0.176 | 0.068 | 1.000 |  |  |  |  |  |
| Living area | 0.044 | 0.036 | -0.072 | -0.170 | 1.000 |  |  |  |  |
| region | 0.055 | -0.015 | -0.001 | 0.005 | -0.116 | 1.000 |  |  |  |
| Ethnic group | 0.003 | 0.057 | -0.026 | -0.048 | 0.022 | 0.042 | 1.000 |  |  |
| Education level | -0.185 | -0.096 | 0.302 | -0.180 | -0.293 | 0.043 | -0.082 | 1.000 |  |
| Chronic diseases | 0.283 | -0.060 | -0.038 | 0.118 | -0.120 | -0.039 | 0.120 | 0.002 | 1.000 |

Supplementary table 2 Normality Test (P<0.05)

| Variables | Obs | Pr (Skewness) | Pr (Kurtosis) | Joint P-Value |
| --- | --- | --- | --- | --- |
| age | 466 | 0.302 | 0..009 | 0.022 |
| Living area | 466 | 0.000 | 0.884 | 0.000 |
| Ethnic group | 466 | 0.000 | 0.000 | 0.000 |
| Education level | 466 | 0.705 | 0.000 | 0.000 |
| Chronic diseases | 466 | 0.000 | 0.001 | 0.000 |
